# Cutaneous metastasis of breast carcinoma with an angiokeratoma-like pattern: a dermoscopic diagnostic pitfall^؆^

**DOI:** 10.1016/j.abd.2026.501450

**Published:** 2026-08-29

**Authors:** Juliane da Costa Araujo, Eduardo Lise Perin, Mateus Ceolin Vione, Leandro Linhares Leite

**Affiliations:** a Department of Dermatology, Hospital de Clínicas de Porto Alegre, Porto Alegre, RS, Brazil; b Department of Pathology, Hospital de Clínicas de Porto Alegre, Porto Alegre, RS, Brazil

Dear Editor,

A 37-year-old female patient sought dermatological care for asymptomatic, gradually progressive skin lesions on her right breast that had been present for approximately five months. Her past medical history included invasive breast carcinoma of the right breast (stage T4dN+[N+]M1 with hepatic metastasis), diagnosed three years prior; the tumor was estrogen receptor-negative. She had also had cerebellar metastases treated with radiotherapy. At the time, she was undergoing immunotherapy with trastuzumab and pertuzumab.

Dermatological examination revealed multiple well-defined, reddish-blue papules clustered on the right breast, with a clinical appearance suggestive of angiokeratomas (Fig. 1).

**Figure 1:**
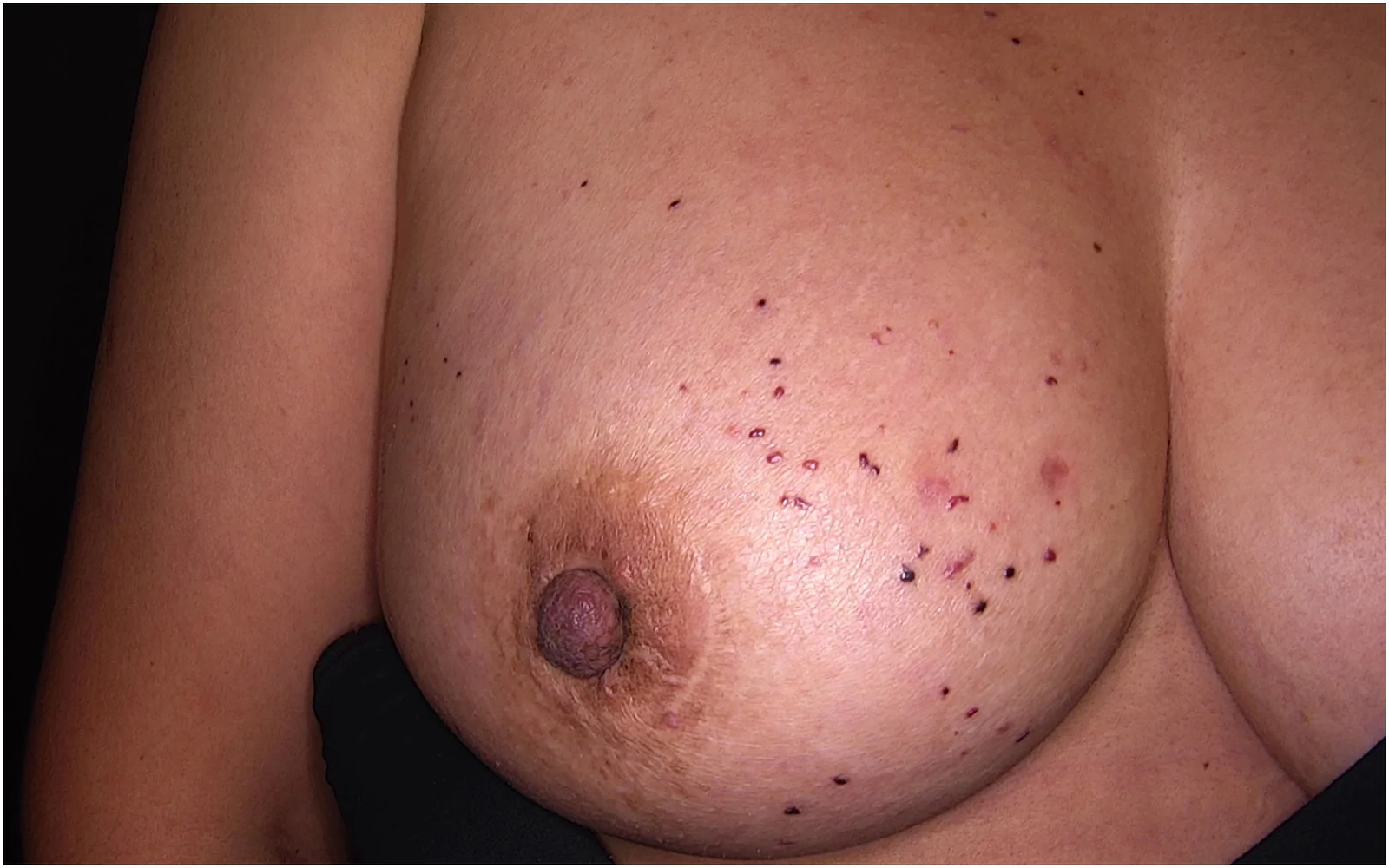
**Clinical appearance of the cutaneous lesions.** Multiple erythematous-violaceous to reddish-black papules of varying sizes—some with a hemorrhagic appearance—clustered on the right breast, clinically mimicking angiokeratomas and corresponding to cutaneous metastases of breast carcinoma.

Dermoscopy showed well-defined reddish-blue lacunae associated with a whitish veil, without evident polymorphic vessels—a classic pattern described for angiokeratoma—representing a potential diagnostic pitfall^1^ (Fig. 2).

**Figure 2:**
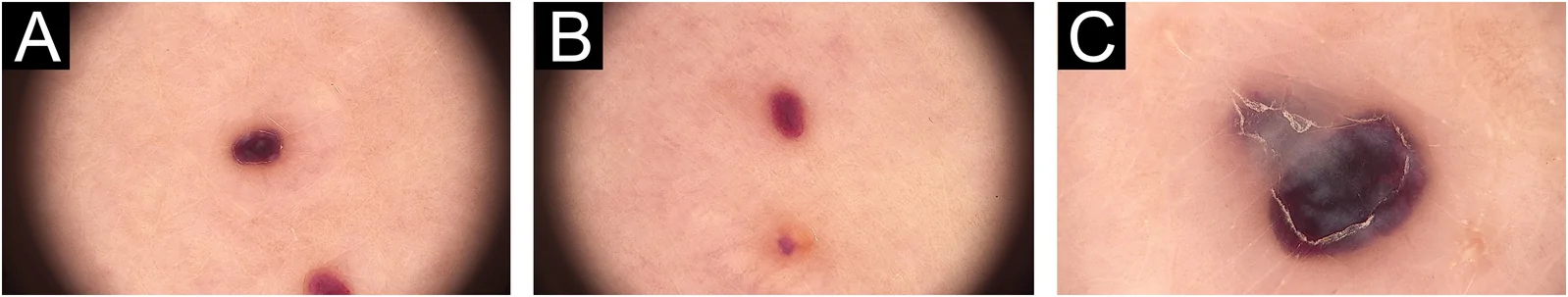
**Dermoscopy of skin lesions on the right breast.** (A) Well-demarcated violaceous papule exhibiting homogeneous red-blue lacunae. (B) Smaller violaceous papular lesion without polymorphic vascular structures. (C) Larger lesion with blue-black areas associated with a superficial whitish veil, mimicking the dermoscopic pattern of an angiokeratoma.

Histopathological analysis revealed vascular embolization by an undifferentiated malignant neoplasm. Immunohistochemistry showed positivity for CK7, mammaglobin, GATA-3, HER2, podoplanin, and CD31; these findings were consistent with cutaneous metastasis of breast carcinoma with lymphatic embolization (Fig. 3).

**Figure 3:**
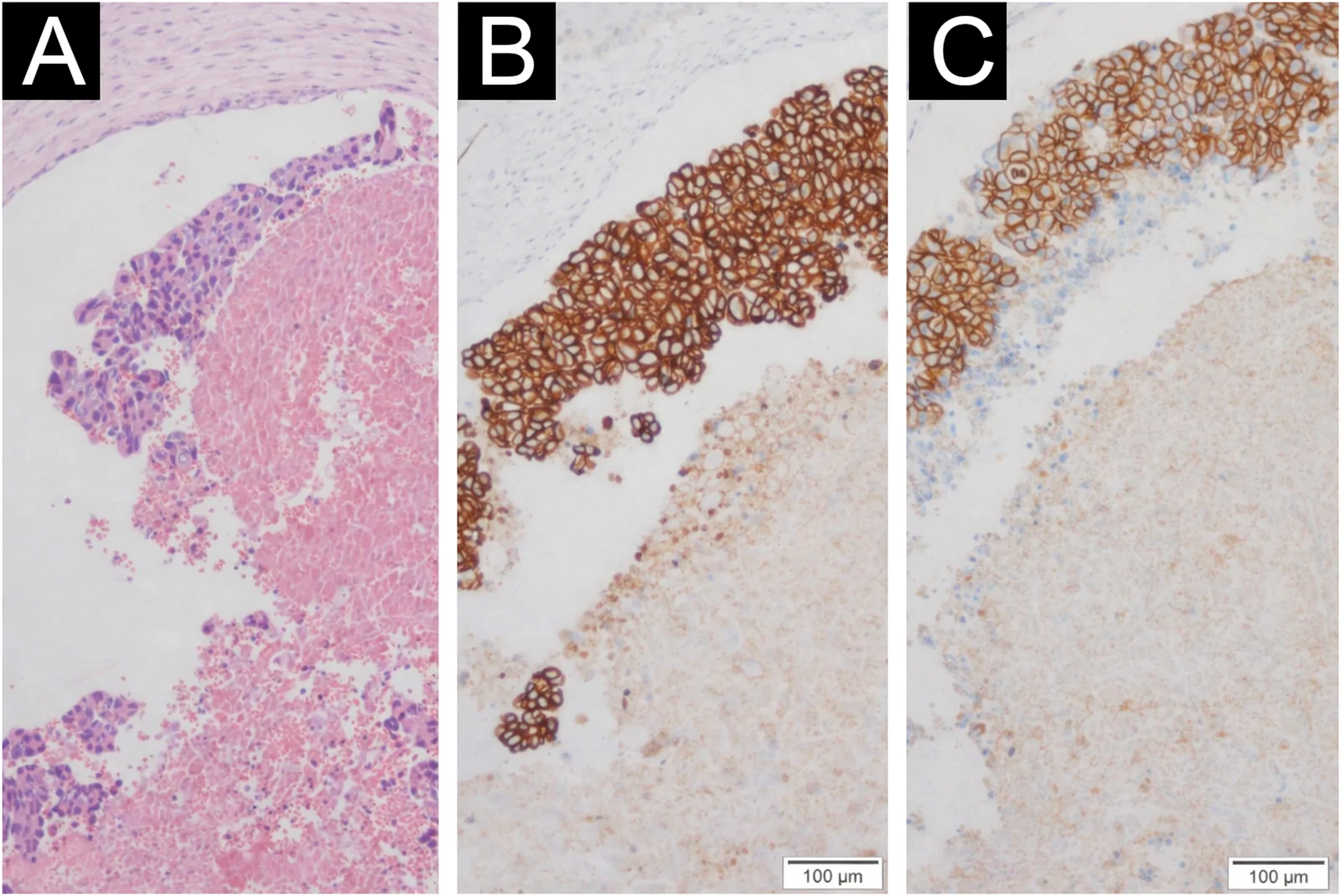
**Histopathological and immunohistochemical study of the cutaneous lesion.** (A) Histological section stained with Hematoxylin & Eosin (H&E), showing dermal vascular embolization by aggregates of neoplastic cells associated with extravasated blood (×100 magnification). (B) Immunohistochemical study showing diffuse cytoplasmic positivity for CK7 in tumor cells, confirming an epithelial origin (×100 magnification). (C) Immunohistochemistry demonstrating HER2 overexpression in neoplastic cells, consistent with cutaneous metastasis of HER2-positive breast carcinoma (×100 magnification).

## Discussion

Cutaneous metastases represent a relatively uncommon yet clinically relevant manifestation of internal malignancy spread, generally associated with advanced disease and a poorer prognosis.^2^ Among women, breast cancer is the primary source of these lesions, frequently affecting the anterior chest and the breast, with varied and potentially misleading clinical presentations.^3^

Dermoscopy has been described as an adjunctive tool in the evaluation of cutaneous metastases. Studies show that non-melanocytic metastases—including those from breast cancer—are predominantly characterized by structureless white areas associated with linear serpiginous vessels, as well as polymorphic and disorganized vessels.^4^, ^5^, ^6^ However, there are no definitive dermoscopic criteria, and the variability of findings limits diagnostic accuracy.^5^, ^6^

In the present case, an angiokeratoma-like dermoscopic pattern was observed, characterized by red-blue lacunae and a whitish veil, without polymorphic vessels—findings typical of benign vascular lesions.^1^ This pattern contrasts with that described in large multicenter series, where well-defined hemorrhagic lacunae are rarely observed in cutaneous metastases, underscoring the atypical and misleading nature of the observed presentation.^6^

The mimicry of benign lesions by cutaneous metastases is well documented, leading these manifestations to be considered true "great imitators" in dermatology.^3^ In breast cancer, atypical vascular presentations may be related to lymphatic or vascular embolization by tumor cells, explaining the dermoscopic findings observed in this case.^5^

This report highlights the limitations of dermoscopy when used in isolation and reinforces the need to maintain a high index of suspicion regarding new, progressive, or atypical lesions in cancer patients. Even when dermoscopy strongly suggests a benign lesion, a skin biopsy remains mandatory to establish the diagnosis.^4^, ^5^, ^6^

In conclusion, we describe a cutaneous metastasis of breast carcinoma with an angiokeratoma-like pattern—clinically and dermoscopically misleading—highlighting the importance of recognizing such unusual presentations and the fundamental role of histopathology in confirming the diagnosis.

## ORCID ID

Eduardo Lise Perin: 0009-0004-8774-7242

Mateus Ceolin Vione: 0009-0005-1659-6421

Leandro Linhares Leite: 000-0001-6370-3115

## Research data availability

Does not apply.

## Financial support

None declared.

## Authors' contributions

Juliane da Costa Araujo: Design and planning of the study; collection, analysis, and interpretation of data; intellectual participation in the propaedeutic and/or therapeutic conduct of the studied case; drafting and editing of the manuscript; critical review of the literature; approval of the final version of the manuscript.

Eduardo Lise Perin: Collection, analysis, and interpretation of data; intellectual participation in the propaedeutic and/or therapeutic conduct of the studied case; drafting and editing of the manuscript; critical review of the literature; approval of the final version of the manuscript.

Mateus Ceolin Vione: Collection, analysis, and interpretation of anatomopathological data and provision of images.

Leandro Linhares Leite: Effective participation in research orientation; intellectual participation in the propaedeutic and/or therapeutic conduct of the studied case; critical review of the manuscript; approval of the final version of the manuscript.

## Conflicts of interest

None declared.
